# Enhanced Recovery After Surgery (ERAS) in Cardiac Surgery: From Evidence to Precision Perioperative Medicine

**DOI:** 10.3390/jcm15218582

**Published:** 2026-11-04

**Authors:** Isidro Olavide, Pablo Montero-López, Marc Vives

**Affiliations:** Department of Anesthesiology and Critical Care, Clínica Universidad de Navarra, 31008 Pamplona, Spain

**Keywords:** enhanced recovery after surgery, ERAS, cardiac surgery, perioperative medicine, enhanced recovery, frailty, prehabilitation, patient blood management, goal-directed perfusion, functional recovery

## Abstract

Cardiac surgery is increasingly performed in older, frailer patients with multimorbidity, making postoperative recovery dependent not only on technical success but also on the quality of perioperative care. Enhanced Recovery After Surgery (ERAS) programs address this challenge through coordinated preoperative optimization, attenuation of surgical stress, organ protection, opioid-sparing analgesia, early mobilization, and structured discharge. This narrative review critically appraises ERAS in cardiac surgery and organizes the evidence across the perioperative pathway. Available studies associate complete ERAS programs with modest reductions in intensive care and hospital length of stay and possible reductions in overall complications, but evidence certainty is limited by heterogeneous bundles, single-center designs, and predominance of observational studies. Component-level evidence is strongest or clinically most relevant for frailty assessment, Patient Blood Management, multimodal analgesia, oxygen delivery-guided perfusion, delirium prevention, and early mobilization, although universal thresholds and isolated technologies should be avoided. Successful implementation depends on multidisciplinary governance, explicit responsibilities, adherence measurement, audit, and patient-centered outcomes. Future development should retain a standardized core while allowing transparent adaptation to baseline risk and observed response. Precision perioperative approaches remain hypothesis-generating and require external validation, clinical impact evaluation, assessment of equity, and demonstration of cost-effectiveness before routine adoption.

## 1. Introduction

Advances in surgery, cardiovascular anesthesia, cardiopulmonary bypass, and critical care have reduced operative mortality and broadened the indications for cardiac surgery. Consequently, older patients with greater frailty, anemia, renal dysfunction, diabetes, malnutrition, and functional impairment are increasingly undergoing surgery. In this setting, outcomes no longer depend solely on technically correcting the cardiac disease; they also depend on how the patient is prepared, the magnitude of avoidable perioperative insults, the prevention of organ injury, and the system’s ability to restore function and independence promptly. Cardiac surgery-specific ERAS recommendations published in 2019 and updated in 2024, together with European and national guidance, have consolidated this cross-cutting approach [1,2,3,4].

The concept did not emerge abruptly. Early fast-track trials showed that early extubation was feasible without increasing morbidity in selected patients [5]. A subsequent randomized trial evaluated a broader ERAS pathway [6]. Meta-analyses of trials and observational studies have reported shorter lengths of stay and signals of fewer complications, but have also documented heterogeneity, risk of bias, and low certainty [7,8]. Published in 2026, the INCREASE trial provides recent randomized evidence for an interdisciplinary, cross-sectoral model in minimally invasive heart valve surgery, although its setting and population limit automatic extrapolation to cardiac surgery as a whole [9].

Cardiac surgery poses specific challenges for ERAS. Cardiopulmonary bypass, ischemia–reperfusion, hemodilution, anticoagulation, bleeding, transfusion, and mechanical ventilation interact with highly variable physiological reserve. Published programs also differ in patient selection, number and content of components, definitions of adherence, and outcomes. The same label encompasses extubation protocols, analgesic bundles, comprehensive pathways, and organizational improvement projects. This variability explains why the overall effect cannot be used to infer the efficacy of each individual intervention.

This review aims to critically appraise the evidence on ERAS in cardiac surgery and organize it into a coherent clinical sequence. It first describes the evolution of fast-track care. It then integrates physiological rationale, clinical evidence, and practical application within the preoperative, intraoperative, and postoperative phases. Finally, it addresses implementation, methodological controversies, and the possible transition toward more personalized perioperative medicine. The objective is not to turn every reasonable practice into a strong recommendation, but to distinguish clearly between what has been demonstrated, what is plausible, and what remains uncertain.

### Literature Search and Review Approach

This narrative review was informed by searches of PubMed/MEDLINE and Google Scholar from database inception to 5 August 2026. The search combined controlled vocabulary and free-text terms related to “Enhanced Recovery After Surgery”[MeSH], “Cardiac Surgical Procedures”[MeSH], “Cardiopulmonary Bypass”[MeSH], “Perioperative Care”[MeSH], “Frailty”[MeSH], “Exercise Therapy”[MeSH], “Patient Blood Management”[MeSH], “Analgesia”[MeSH], “Acute Kidney Injury”[MeSH], “Delirium”[MeSH], and “Early Ambulation”[MeSH], together with the terms cardiac surgery, fast-track, prehabilitation, multimodal analgesia, goal-directed perfusion, cerebral oximetry, and enhanced recovery. Reference lists of relevant guidelines, consensus statements, trials, and systematic reviews were also screened. Priority was given to society guidelines, randomized trials, systematic reviews and meta-analyses, and prospective cohorts directly involving adult cardiac surgery. Because no preregistered protocol, duplicate study selection, or formal risk-of-bias assessment was undertaken, this work should be interpreted as a narrative review rather than a systematic review.

A targeted supplementary literature search was conducted on 21 October 2026 using the additional terms “multimorbidity”, “multiple chronic conditions”, “shared decision-making”, and “patient preferences” in combination with cardiac surgery, perioperative care, and older adults. PubMed-indexed literature and relevant cardiovascular and perioperative reviews identified through online scholarly searches were screened for conceptual and clinical relevance. This targeted update informed the distinction between frailty and multimorbidity and the discussion of patient goals; it was not a systematic search or a formal evidence synthesis [10].

## 2. From Fast-Track Care to an Integrated ERAS Pathway

Early fast-track programs focused on reducing anesthetic and opioid doses, facilitating emergence, and accelerating extubation. The trial by Cheng and colleagues showed that an early-extubation strategy after coronary surgery could reduce ventilation time and ICU and hospital length of stay without increasing morbidity [5]. This finding was important because it challenged routine prolonged ventilation, but it did not yet constitute a comprehensive recovery program. Extubation is an intermediate outcome that leaves many variables unexamined.

ERAS broadened the focus from a respiratory milestone to the entire care pathway. The 2019 recommendations organized preoperative, intraoperative, and postoperative interventions and formalized the role of the multidisciplinary team [2]. The 2024 consensus strengthened pathway-specific coordination, adherence measurement, and continuous improvement [1]. The essential difference is that ERAS does not seek merely to optimize process times, but to reduce avoidable variability through coordinated decisions, shared goals, and frequent reassessment.

**Figure 1:**
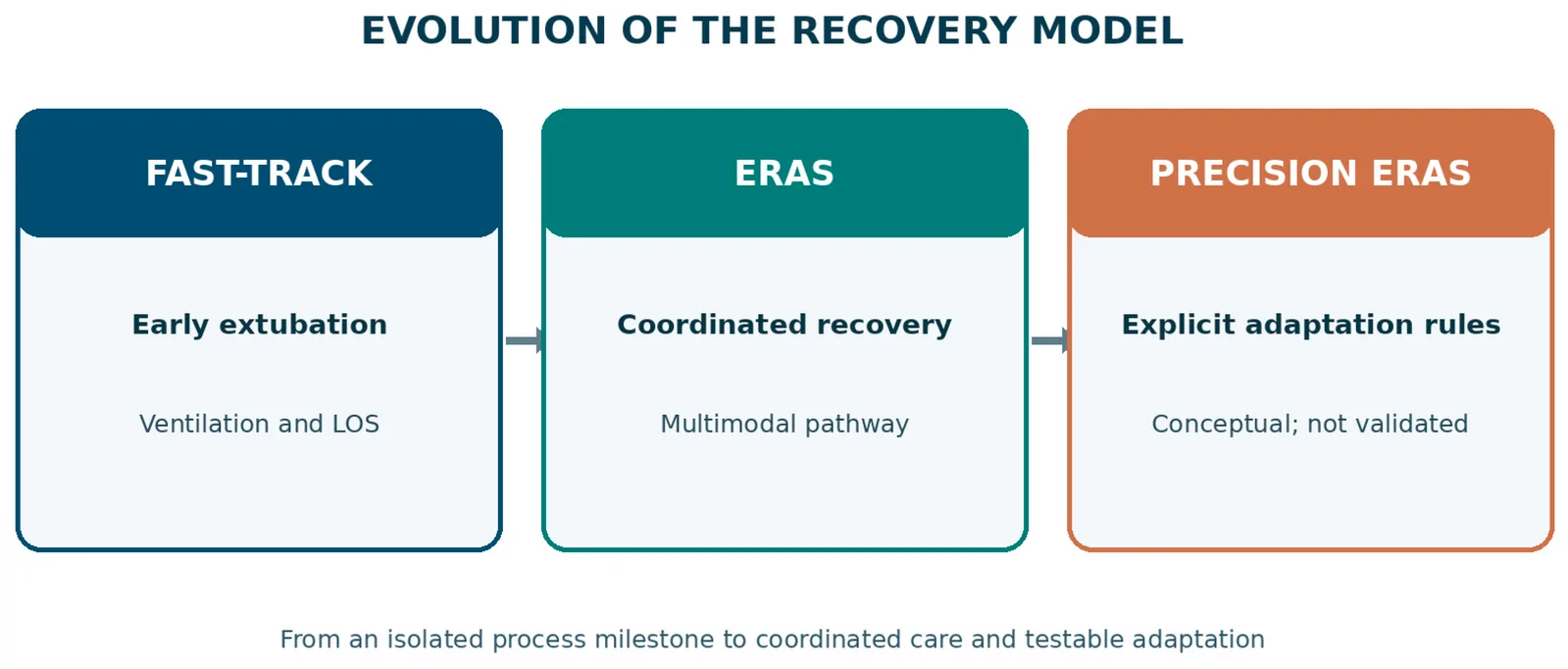
Simplified evolution of recovery models in cardiac surgery: fast-track care focuses on early extubation, ERAS coordinates a multimodal recovery pathway, and proposed precision ERAS makes adaptation rules explicit. Precision ERAS remains conceptual and requires prospective validation.

Figure 1 places this evolution in context. The transition from fast-track care to precision ERAS should not be interpreted as one model replacing another, but as a progressive expansion of the objective: from early emergence to multimodal recovery and, ultimately, adaptation of intervention intensity to the patient’s risk and response.

This expansion creates a methodological tension: the more comprehensive the bundle, the harder it becomes to attribute benefit to a specific component. Part of the effect may derive from clinical interventions, while another part may arise from standardization, communication, anticipation of problems, and audit. ERAS should therefore be understood simultaneously as a set of practices and an organizational model.

## 3. ERAS Recommendations and Contemporary Evidence Across the Perioperative Pathway

The 2019 ERAS Society recommendations and the 2024 joint consensus provide the clinical framework for this section [1,2]. Each subsection states the ERAS position, examines the primary studies and evidence syntheses that support or qualify it, and ends with a clinical interpretation. Recovery is considered as a sequence in which baseline physiological reserve, the surgical and cardiopulmonary-bypass insult, organ-protection strategies, and restoration of function remain clinically connected.

**Figure 2:**
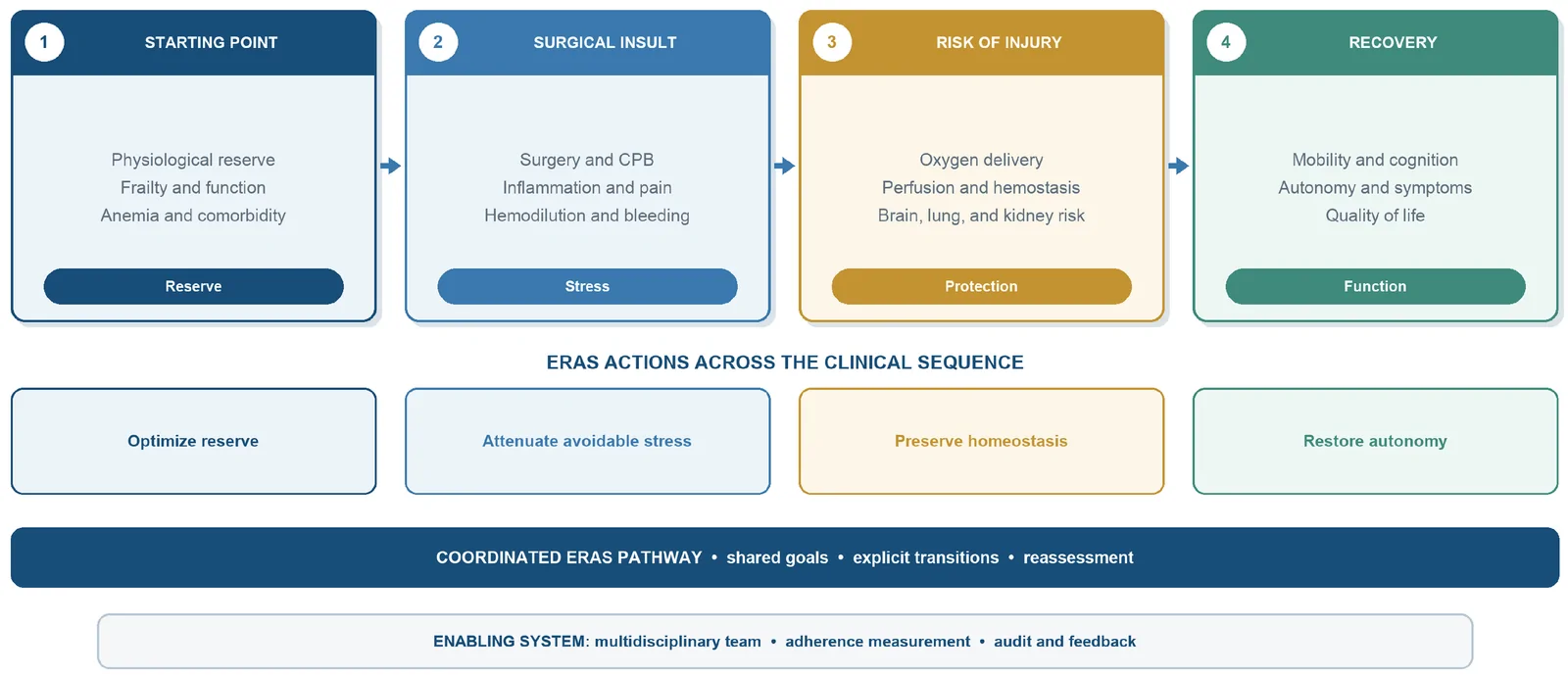
Conceptual sequence used to organize the integrated appraisal of ERAS in cardiac surgery: baseline reserve, surgical insult, risk of organ injury, and functional recovery. Interventions may act on more than one link and require cross-cutting coordination.

Figure 2 summarizes this sequence. The blocks are interconnected: a single intervention may act at several time points. Patient Blood Management begins with diagnosing anemia, continues with reducing blood loss, and affects physiological tolerance during perfusion; similarly, analgesia limits nociceptive stress and subsequently facilitates ventilation and mobilization.

### 3.1. Overall Evidence for Integrated ERAS Pathways

**ERAS position.** The 2019 recommendations and 2024 joint consensus define enhanced recovery as a coordinated multidisciplinary pathway rather than a single intervention; the 2024 statement also emphasizes a dedicated team, adherence auditing, and outcome feedback as core program features [1,2].

**Evidence and limitations.** Appraisal of the overall evidence requires study designs to be considered separately. A meta-analysis of 13 single-center trials of “ERAS-like” protocols included 1704 patients and found shorter ICU and hospital stays and fewer overall complications, with no significant difference in mortality [7]. However, the bundles did not necessarily reproduce the complete ERAS recommendations, and the reduction in complications was driven by selected outcomes and studies.

The meta-analysis by Diz-Ferreira and colleagues included 18 studies and 4469 patients, only one of which was randomized. ERAS was associated with a 1.24-day shorter hospital stay, with no differences in mortality or atrial fibrillation; certainty was rated very low, and quality-of-life data were too sparse and heterogeneous to yield a pooled estimate [8]. The INCREASE trial randomized 201 patients at two centers performing minimally invasive heart valve surgery. Its prespecified analyses did not demonstrate superiority for hospital days or non-inferiority for the adjusted six-minute walk test, although exploratory sensitivity analyses favored ERAS; safety was broadly comparable except for more postoperative atrial fibrillation in the ERAS group [9]. Because the pathway evaluated several coordinated measures simultaneously, it cannot identify the contribution of individual components, and its specialized setting limits extrapolation to urgent or highly complex surgery.

**Clinical interpretation.** The data support ERAS as a reasonable strategy for standardization and improvement, but do not justify assuming that any bundle labelled ERAS is effective by definition. Content, adherence, patient population, team experience, and the discharge system all influence outcomes.

Table 1 maps the principal interventions discussed in this review to the evidence supporting them. It distinguishes program-level evidence from component-specific evidence and makes explicit where recommendations rely mainly on consensus or indirect evidence.

### 3.2. Preoperative Recommendations: Baseline Reserve and Optimization

#### 3.2.1. Education, Risk Stratification, Frailty, and Prehabilitation

**ERAS position.** The 2024 consensus supports multifaceted screening and risk assessment for perioperative planning (moderate-quality evidence), shared decision-making and patient engagement (low-quality evidence), and consideration of multicomponent prehabilitation before nonurgent cardiac surgery (low-quality evidence) [1].

**Evidence and limitations.** Preoperative education should explain the expected pathway, pain management, respiratory and mobilization goals, patient participation, and discharge criteria. A systematic review and meta-analysis of 22 randomized trials including 3167 cardiac surgery patients found improved preoperative knowledge, less perioperative anxiety, and greater satisfaction, although the interventions and clinical outcomes were heterogeneous [11]. Education should therefore be evaluated not only by whether information was delivered, but also by whether the patient understood the recovery goals and was able to participate in them.

Physiological reserve describes the patient’s ability to tolerate surgical stress and restore homeostasis. Frailty identifies a reduction in this reserve but is not synonymous with age, disability, or comorbidity, although it may coexist with them. The Fried phenotype operationalized frailty across five domains: unintentional weight loss, exhaustion, weakness, slow walking speed, and low physical activity; three or more criteria define frailty, whereas one or two define prefrailty [12]. Other instruments, including the Clinical Frailty Scale, the Essential Frailty Toolset, and deficit-accumulation indices, measure partly different dimensions. There is therefore no universal instrument or interchangeable threshold.

Frailty and multimorbidity are related but non-interchangeable dimensions of risk. Multimorbidity describes the coexistence of two or more chronic conditions, whereas frailty reflects diminished physiological reserve and vulnerability to stressors. A patient may have multimorbidity without frailty, or frailty without extensive multimorbidity. Preoperative evaluation should therefore consider both domains rather than treating comorbidity counts as a proxy for frailty. These constructs should be recorded separately when planning care; prognostic associations alone do not establish that modifying either construct improves outcomes.

In a prospective cohort of 529 cardiac surgery patients, frail patients had longer ICU and hospital stays and higher unadjusted in-hospital mortality; after adjustment, the mortality estimate was imprecise and did not reach statistical significance [13]. A meta-analysis of 62 studies including 16,679 patients confirmed associations between frailty and mortality, complications, institutionalization, readmission, and poorer quality of life [14]. These associations provide prognostic information, but they do not demonstrate that all frailty is reversible or that improving a scale score alone leads to better outcomes.

Prehabilitation seeks to increase reserve before surgery through exercise, inspiratory muscle training, nutrition, and psychological support. A meta-analysis of six studies including 665 patients found improved six-minute walking distance and an approximately 1-day reduction in length of stay with exercise-based programs, although heterogeneity and small study size limit certainty [15]. Feasibility is conditioned by the time available before surgery, urgency, adherence, and patient selection; the optimal combination, minimum duration, and incremental benefit in already active patients remain unknown.

**Clinical interpretation.** Frailty assessment is useful only if it changes the plan. The preoperative visit should produce a written plan that identifies modifiable domains, defines a feasible prehabilitation strategy, anticipates ICU and rehabilitation needs, and records responsible professionals and time frames. Measuring frailty or documenting education without linking the result to care decisions adds burden but not value.

Shared decision-making should connect multidisciplinary risk assessment with the outcomes that matter most to the patient. Before recommending a pathway, the team should consider likely benefits, competing treatment options, anticipated complications, and realistic trajectories of functional and cognitive recovery. These estimates should be discussed with the patient in relation to personal priorities, such as survival, independence, symptom relief, and acceptable trade-offs.

#### 3.2.2. Nutritional and Metabolic Preparation

**ERAS position.** Current ERAS guidance includes nutritional risk within preoperative assessment and allows nutrition to form part of multicomponent prehabilitation. It does not provide cardiac-specific support for routine carbohydrate loading in every patient; permitting clear liquids until two hours before surgery may be reasonable only after aspiration-risk assessment, with low-quality evidence [1,2].

**Evidence and limitations.** Preoperative nutritional assessment should be treated as risk stratification rather than as an isolated laboratory evaluation. In a single-center retrospective cohort of 197 patients undergoing open-heart surgery with cardiopulmonary bypass, the Malnutrition Universal Screening Tool (MUST) independently predicted postoperative decline in activities of daily living (adjusted OR 4.75; 95% CI 1.37–16.5) [16]. Severe complications and longer hospital stay were associated with nutritional-risk measures in unadjusted analyses but were not demonstrated as independent effects of MUST after adjustment. These findings support systematic screening linked to a feasible intervention, but do not demonstrate that correcting a screening score improves outcomes.

Cardiac surgery trials of preoperative carbohydrate loading show acceptable safety in selected elective patients but inconsistent metabolic and clinical benefits. In ASA III-IV patients, carbohydrate administration did not reduce postoperative insulin resistance or hospital stay, whereas clear fluids reduced thirst without increasing gastric volume or reported adverse events [17]. In off-pump coronary artery bypass grafting, insulin sensitivity improved after induction, but the difference was no longer present after surgery [18]. A more recent on-pump trial in nondiabetic adults found no significant effect on perioperative glucose concentrations or HOMA-IR [19]. Carbohydrate loading should therefore not be presented as a universal cardiac surgery intervention and should be individualized according to diabetes, gastric emptying, heart failure, aspiration risk, and scheduling reliability.

**Clinical interpretation.** The preoperative plan should document nutritional risk, the fasting and hydration strategy, relevant contraindications, and the intervention selected. Useful indicators include completion of nutritional screening, cancellations or delays related to preparation, and adverse events rather than carbohydrate administration alone.

#### 3.2.3. Anemia and Patient Blood Management

**ERAS position.** ERAS guidance recommends a comprehensive Patient Blood Management program spanning preoperative anemia and iron-deficiency screening and treatment when indicated, minimization of blood loss and hemodilution, and standardized restrictive rather than liberal transfusion strategies; the 2024 consensus assigns moderate quality to the program-level statement [1,2].

**Evidence and limitations.** Among 4117 patients undergoing cardiac surgery, preoperative anemia was associated with acute kidney injury and longer length of stay. Mediation analysis showed that most of the association with kidney injury was not explained by intraoperative transfusion [20]. This association does not establish causality, but it supports early etiological assessment rather than treating transfusion as a substitute for diagnosing anemia.

Patient Blood Management begins before admission and integrates preoperative diagnosis and treatment, reduction in blood loss, guided hemostasis, and context-specific transfusion decisions [22]. A complete blood count and etiological evaluation should be performed early enough to address iron deficiency or other treatable causes. In a randomized trial of 505 patients with anemia or isolated iron deficiency, a combination of intravenous iron, erythropoietin alpha, vitamin B12, and folic acid administered the day before surgery reduced red-cell and total allogeneic transfusions [21]. Because the intervention was bundled and conducted at a single center, it does not establish the independent efficacy of iron or erythropoietin and should not justify empirical treatment without etiological assessment.

TITRe2 showed that a restrictive strategy reduces transfusion exposure but detected a signal of higher 90-day mortality that argues against simplistic interpretations [23]. The economic analysis of the same trial and a subsequent meta-analysis likewise do not support a policy based solely on cost or a fixed threshold [24,25]. Decisions should consider active bleeding, ischemia, oxygenation, cardiopulmonary reserve, and response to alternative measures.

**Clinical interpretation.** Indicators should encompass anemia detected, investigated, and treated before surgery; transfusion by phase of care; exposure to blood components; re-exploration for bleeding; and complications, not merely the proportion of patients crossing a hemoglobin threshold.

### 3.3. Intraoperative Recommendations: Modulation of Stress and Organ Protection

Cardiac surgery activates neuroendocrine, inflammatory, metabolic, and hemostatic responses. Cardiopulmonary bypass adds blood contact with artificial surfaces, hemodilution, temperature changes, and ischemia–reperfusion. These pathways may lead to insulin resistance, catabolism, endothelial dysfunction, microcirculatory abnormalities, edema, and organ injury. This is not a single mechanism, and no intervention can neutralize it completely.

ERAS measures act on different mechanisms and at different times. Analgesia reduces nociceptive and sympathetic stimulation; normothermia prevents the consequences of hypothermia; risk-adjusted ventilation limits additional lung injury; and management of fluids, hemodilution, bleeding, and perfusion reduces edema, coagulopathy, and impaired oxygen delivery [1,2,3,4]. Thus, attenuating the stress response means preventing avoidable secondary insults and their consequences, not abolishing the physiological response required for surgery.

#### 3.3.1. Anesthesia, Multimodal Analgesia, and Regional Techniques

**ERAS position.** The 2024 consensus supports multimodal opioid-sparing analgesia and chest-wall regional analgesia as components of perioperative pain management, both with moderate-quality evidence, while recognizing that drug and block choices must be individualized [1].

**Evidence and limitations.** The anesthetic strategy should provide hemodynamic stability, timely and assessable emergence, and pain control without pursuing extubation at the expense of safety. In a trial of 151 patients, a multimodal regimen reduced pain, nausea, and vomiting compared with opioid-based analgesia, although patient selection and the drug combination limit generalizability [26]. The objective is not an opioid-free practice but reduced unnecessary exposure while maintaining sufficient analgesia for spontaneous ventilation and mobilization.

Parasternal fascial-plane blocks may be incorporated as an adjunct to multimodal analgesia after median sternotomy. A systematic review of 11 randomized trials including 756 patients found a modest reduction in 24-h opioid consumption with superficial parasternal intercostal plane blocks, but the prespecified 12-h outcome was reported by only one trial and safety data covered only 196 blocks [27]. Current evidence therefore supports a possible opioid-sparing effect but does not establish superiority among block types or justify a universal technique. Selection should consider surgical approach, local expertise, antithrombotic therapy, and monitoring for local anesthetic systemic toxicity.

**Clinical interpretation.** Pain at rest and with movement, morphine-equivalent consumption, nausea, sedation, respiratory function, and adverse events should be recorded. Low opioid use is not a successful outcome if accompanied by pain that prevents breathing exercises or mobilization. Regional anesthesia is feasible, and it may be adapted to local protocols and experience.

#### 3.3.2. Hemodynamics, Cardiopulmonary Bypass, Oxygen Delivery, and Organ Protection

**ERAS position.** The 2024 consensus states that goal-directed perfusion may help prevent organ injury associated with cardiopulmonary bypass but rates the evidence as low. It considers central nervous system monitoring potentially useful for detecting neurological risk, with moderate-quality evidence and uncertainty about the optimal corrective algorithm [1].

**Evidence and limitations.** One link between perioperative stress and organ injury is an imbalance between oxygen delivery and consumption. Arterial oxygen content, pump flow, hemodilution, temperature, bleeding, and transfusion influence oxygen delivery and tissue perfusion, but they are not equivalent interventions and should not be reduced to a single target.

During cardiopulmonary bypass, arterial oxygen content and pump flow determine indexed oxygen delivery, which is also modulated by temperature. In Ranucci’s multicenter trial, a strategy targeting a DO_2_i of at least 280 mL/min/m^2^ reduced AKIN stage 1, but not AKIN stages 2–3, mortality, transfusion, or length of stay [28]. In the single-center randomized trial by Mukaida et al., 300 patients were assigned to a DO_2_-guided strategy targeting DO_2_i > 300 mL/min/m^2^ or conventional fixed-flow perfusion; among 275 analysed patients, postoperative AKI occurred in 14.6% versus 30.4%, respectively (RR 0.48; 95% CI 0.30–0.77) [29]. The updated European guideline integrates flow, pressure, venous saturation, DO_2_, lactate, temperature, and neuromonitoring as complementary signals [30]. DO_2_ should therefore be interpreted alongside hemodilution, bypass duration, thermal strategy, pressure, lactate, and renal reserve rather than as an isolated universal target.

Regional cerebral oxygen saturation measured by near-infrared spectroscopy (NIRS) can detect disturbances associated with hypotension, low flow, hypoxemia, hypocapnia, hemodilution, cannula position, or impaired venous drainage [30]. A 2024 meta-analysis of randomized trials found less delirium with NIRS-guided algorithms, but no differences in ICU length of stay or renal or cardiac outcomes [31]. When NIRS is used, the center should define a relative or absolute threshold and a stepwise intervention sequence; the monitor alone does not guarantee prevention of neurological injury.

**Clinical interpretation.** Organ protection requires joint interpretation of hemoglobin, flow, DO_2_, pressure, temperature, bleeding, lactate, and perfusion signals. An abnormality should prompt confirmation of its cause and stepwise correction; neither NIRS nor an isolated DO_2_ value is, by itself, an indication for transfusion.

#### 3.3.3. Ventilation, Temperature, Glycemic Control, and Fluid Therapy

**ERAS position.** ERAS guidance supports lung-protective ventilation, normothermia, avoidance of clinically important hyperglycemia, and goal-directed hemodynamic and fluid management. The 2024 consensus assigns high quality to lung-protective ventilation, considers ventilation during cardiopulmonary bypass uncertain, and supports goal-directed resuscitation with moderate-quality evidence [1,2].

**Evidence and limitations.** Ventilation, temperature, glycemic control, and fluid therapy should be individualized rather than represented by fixed universal targets. In the multicenter PROVECS trial, an open-lung strategy combining ventilation during cardiopulmonary bypass, recruitment maneuvers, and higher PEEP did not reduce postoperative pulmonary complications compared with conventional ventilation [32]. This result does not negate low-tidal-volume ventilation but shows that adding a complete open-lung package is not beneficial by definition. Thermal management should avoid both hypothermia-related coagulopathy and excessive rewarming and should be integrated with the overall perfusion strategy [30].

For glycemic control, the GLUCO-CABG trial found no overall reduction in a composite of perioperative complications with an intensive target of 100–140 mg/dL compared with 141–180 mg/dL [33]. The objective should therefore be prevention of persistent hyperglycemia without increasing hypoglycemia, rather than normalization at any cost. Fluid composition alone is also insufficient: in the 1136-patient LICRA trial, a chloride-restricted perioperative fluid strategy did not reduce acute kidney injury [34]. Fluid decisions should integrate volume responsiveness, congestion, hemodilution, renal function, and perfusion.

**Clinical interpretation.** Each measure should be linked to the disturbance it is intended to prevent and to a corresponding indicator: respiratory mechanics and gas exchange for ventilation; temperature for thermal management; glucose and hypoglycemia for glycemic control; and fluid balance, hemodilution, congestion, and perfusion for fluid therapy. Adherence to an isolated target does not compensate for an important disturbance that remains undetected or untreated.

#### 3.3.4. Hemostasis and Blood Conservation

**ERAS position.** Both ERAS documents place antifibrinolysis, minimized hemodilution and blood loss, cell salvage, and algorithm-guided hemostatic therapy within comprehensive Patient Blood Management; the 2024 consensus gives the program-level statement moderate-quality evidence [1,2].

**Evidence and limitations.** Reducing bleeding requires a combination of antifibrinolytic therapy, meticulous surgical technique, minimized hemodilution, blood salvage, and defect-directed hemostatic treatment. In ATACAS, involving 4631 patients undergoing coronary artery surgery, tranexamic acid reduced transfusion and reoperation for major bleeding or tamponade, without reducing the composite of death or thrombotic complications, and increased postoperative seizures from 0.1% to 0.7% [35]. Dose selection and neurological risk therefore require explicit consideration; an antifibrinolytic should not be considered risk-free.

Point-of-care hemostatic testing is useful when embedded in a treatment algorithm rather than used as an isolated device. In a 12-center stepped-wedge cluster-randomized trial including 7402 patients, an integrated point-of-care algorithm reduced platelet transfusion and major bleeding but did not reduce complications or hospital stay [36]. Patient Blood Management connects these measures with preoperative optimization and context-specific transfusion decisions [22].

**Clinical interpretation.** Perfusion, hemostasis, and transfusion should be reviewed together because all three influence oxygen delivery and the risk of organ injury. The protocol should define responses to low DO_2_, bleeding, or anemia and document the probable cause, intervention, and effect. Audit should detect both unnecessary transfusions and persistent episodes of hypoperfusion or uncontrolled bleeding.

### 3.4. Postoperative Recommendations: From Stability to Functional Recovery

Preventing a major complication is necessary, but it is not equivalent to patient recovery. Hemodynamic and respiratory stability may coexist with pain, delirium, weakness, dependence in basic activities, or inability to return home. Relevant outcomes should therefore include physical and cognitive function, independence, symptom control, quality of life, and return to activities, in addition to extubation and length of stay.

#### 3.4.1. Emergence, Extubation, and Respiratory Recovery

**ERAS position.** The 2024 consensus supports structured strategies to facilitate extubation within six hours after elective cardiac surgery (moderate-quality evidence), while reserving intraoperative or immediate extubation for highly selected low-risk patients and experienced programs (low-quality evidence) [1].

**Evidence and limitations.** Early extubation remains a useful objective when the patient meets criteria for hemodynamic stability, ventilation, temperature, hemostasis, and level of consciousness. Fast-track evidence demonstrated feasibility in selected patients [5], but time to extubation should not become the sole target. Failed extubation, reintubation, or delayed recognition of low cardiac output has greater clinical relevance than a modest delay in extubation.

A protocolized emergence strategy should coordinate analgesia, sedation reduction, respiratory physiotherapy, and progressive mobilization while retaining explicit criteria for delaying extubation or escalating support.

**Clinical interpretation.** Time to extubation should be interpreted alongside failed extubation, reintubation, respiratory complications, and the clinical reasons for delay. Early extubation does not offset a preventable reintubation or delayed recognition of hemodynamic deterioration.

#### 3.4.2. Delirium Prevention

**ERAS position.** The 2024 consensus prioritizes routine systematic delirium screening and nonpharmacological prevention, rating this statement as high-quality evidence; pharmacological prophylaxis is not presented as a substitute for a multicomponent bundle [1].

**Evidence and limitations.** Delirium is a multifactorial syndrome. A review of 56 trials found substantial heterogeneity in interventions and definitions [37]. In a pragmatic trial of 331 patients aged 65 years or older, an overnight dexmedetomidine infusion did not reduce delirium and increased hypotension [38]. A subsequent meta-analysis of 32 trials and 6046 participants estimated a reduction in delirium with dexmedetomidine but rated the evidence as low to moderate and retained uncertainty regarding the optimal regimen [39]. This discordance shows that a pooled effect does not validate every dose, timing, or population.

Prevention should begin with risk assessment and a nonpharmacological bundle: orientation, sleep promotion, correction of sensory impairment, analgesia, mobilization, reduced exposure to benzodiazepines and anticholinergics, and treatment of precipitating factors. Pharmacological interventions may be considered in selected contexts, with monitoring for bradycardia and hypotension, but they do not replace these measures.

**Clinical interpretation.** A validated instrument should be used, and delirium-free days, sedative exposure, immobilization, and complications should be recorded. Absence of screening is not evidence of absence of delirium.

For patients who are sufficiently awake and communicative, the Confusion Assessment Method (CAM) or another locally validated instrument may be used; in the ICU, the Confusion Assessment Method for the ICU (CAM-ICU) or the Intensive Care Delirium Screening Checklist (ICDSC) can support structured assessment, including in patients unable to speak. Assessments should be repeated at defined intervals across the postoperative period and after transitions of care, with documentation of sedation level and reasons why assessment is not possible. Instrument selection, staff training, and consistent application matter more than isolated screening.

#### 3.4.3. Nutrition, Device Removal, and Mobilization

**ERAS position.** ERAS pathways encourage restoration of oral intake, early removal of unnecessary devices, and progressive mobilization once safety criteria are met. The 2024 consensus provides moderate-quality support for early ambulation and upper-extremity exercise, whereas cardiac-specific evidence for exact feeding and device-removal timing remains limited [1,2].

**Evidence and limitations.** Functional recovery requires adequate analgesia, management of nausea, restoration of oral intake, sleep, and removal of physical barriers. Adult cardiac surgery evidence for early feeding remains limited. In an 84-patient randomized trial, oral hydration beginning one hour after extubation reduced thirst and improved satisfaction without observed aspiration pneumonia, but the study was not powered to establish safety for rare events [40]. Oral intake should therefore progress according to consciousness, swallowing, hemodynamic and respiratory stability, and gastrointestinal tolerance.

Device removal should likewise follow explicit safety criteria rather than an arbitrary early deadline. In a cluster-randomized trial of 527 patients, chest-tube removal on the day of surgery did not reduce analgesic requirements, improve respiratory function, or reduce complications compared with removal on postoperative day 1, and pleural effusions requiring drainage were frequent in both groups [41]. By contrast, a meta-analysis of 20 studies including 2523 patients associated early mobilization with longer walking distance and shorter hospital and ICU stays, with heterogeneity above 85% and no demonstrated mortality reduction [42]. Mobilization protocols should therefore define dose, progression, safety criteria, and continuity rather than first ambulation alone.

**Clinical interpretation.** Assessment should extend beyond first sitting or ambulation to include independent mobility, walking distance, ability to perform basic activities, and barriers that prevent progression. Low opioid exposure is not a successful outcome if pain prevents breathing exercises or mobilization.

#### 3.4.4. ICU Discharge and Transition Home

**ERAS position.** Contemporary ERAS extends recovery beyond ICU discharge through patient engagement, rehabilitation, and coordinated follow-up. The 2024 consensus encourages prescribed home- or center-based cardiac rehabilitation but does not establish a single transition-home or digital-monitoring model [1].

**Evidence and limitations.** Transition home should be considered an active phase of recovery rather than merely the endpoint of a shorter admission. In a randomized trial of 200 elective cardiac surgery patients, nurse practitioner-led follow-up was associated with fewer symptoms and better physical functioning at two weeks and greater satisfaction at two and six weeks, without a significant reduction in health-care resource use [43]. After coronary artery bypass grafting, the per-protocol analysis of an 88-patient trial found that extending rehabilitation through a mobile application and scheduled nurse contact improved quality of life and functional capacity; rehospitalization was lower, although this estimate was based on only four events in a small single-center study [44]. A subsequent 61-patient pilot trial found a mobile self-management intervention feasible when accessed but did not demonstrate fewer readmissions [45].

Post-discharge monitoring should be targeted to a predefined clinical risk and linked to an explicit response pathway. In the multicenter SEARCH-AF trial, selected patients at high risk of stroke who had no atrial fibrillation documented before surgery or at hospital discharge were randomized to 30-day wearable electrocardiographic monitoring or usual care. Atrial fibrillation or flutter lasting at least six minutes was detected in 19.6% versus 1.7%, respectively [46]. Whether increased detection improves thromboembolic or long-term outcomes remains uncertain.

**Clinical interpretation.** Discharge should require clinical and functional readiness, medication reconciliation, education about warning signs, and a follow-up plan. Outcomes should include discharge destination, independent function, unplanned health-care encounters, readmission, patient-reported recovery, and caregiver burden. Technology should be used only when it addresses a defined risk, is accessible, and triggers an explicit escalation pathway.

## 4. Implementation: From Protocol to Care Delivery System

### 4.1. Team, Leadership, and Governance

Preoperative education, the anesthetic plan, perfusion, analgesia, device removal, nutrition, and mobilization form an ERAS pathway only when they share goals, designated responsibilities, and escalation criteria. Organization is therefore not another biological intervention, but the mechanism that connects all phases of care.

Implementation begins with a team representing all phases of care and being empowered to modify processes. A coordinator should oversee the pathway, identify failures, and connect accountable professionals without replacing the clinical leadership of each discipline. A core set of mandatory interventions, explicit criteria for exclusion or adaptation, and a procedure for reviewing deviations should be defined.

At daily review, the team should agree on what is preventing the patient’s progress and who is responsible for resolving it. Accessible protocols, daily goals, and regular outcome review help maintain continuity across surgery, anesthesia, perfusion, the ICU, nursing, physiotherapy, nutrition, and rehabilitation.

Local adaptation should focus on the objective rather than the specific technique, tailoring evidence-based measures to each center’s capabilities. For example, “pain control that permits mobilization” is an objective, whereas the specific block may vary with expertise and surgical approach. This distinction avoids imposing practices for which a center lacks expertise and facilitates updating the pathway when new evidence emerges.

### 4.2. Adherence, Indicators, and Feedback

Adherence should be measured by component and by patient. A global percentage may conceal that easy measures are completed while higher-impact measures are omitted. Process indicators should be linked to outcomes and safety: education to documented understanding, anemia assessment to treatment, analgesia to pain and mobility, extubation to reintubation, mobilization to function, and discharge to readmission.

Adherence should be interpreted alongside safety and function: early extubation does not offset reintubation, low opioid exposure does not offset disabling pain, and a short stay does not constitute recovery if it increases readmission or post-discharge dependence.

Outcomes should be risk-adjusted and analyzed as time series. Before-and-after comparisons that do not account for changes in selection, technique, bed capacity, or discharge criteria may attribute unrelated organizational effects to the program. Brief, regular feedback is more useful than an annual audit; it should present actionable data and cases in which care deviated from the pathway.

### 4.3. Scale-Up and Sustainability

A staged implementation helps detect unintended effects. It is advisable to begin with a small number of measurable components, establish a baseline, train teams, and then expand the bundle. Sustainability requires embedding responsibilities in routine practice, automating data capture when safe, and reviewing components that do not add value.

Apparent failure may reflect low adherence, an ineffective intervention, a poorly chosen outcome, or an unfavorable context. Each explanation requires a different response. Before adding new measures, the program should demonstrate reliable delivery of existing components and a data system capable of distinguishing adherence, clinical exceptions, and missing documentation.

## 5. Controversies, Evidence Gaps, and Research Priorities

### 5.1. Which Component Produces the Benefit?

Multimodal bundles involve interaction and temporal dependency: analgesia facilitates mobilization; extubation enables communication and feeding; and PBM modifies tolerance of perfusion and transfusion. Conventional trials compare bundles and cannot disentangle these interactions. Factorial, adaptive, and interrupted time-series designs may provide useful information but require large samples and rigorous definitions of adherence.

The contribution of organization is particularly difficult to isolate. A protocol may improve outcomes because it reduces variability, mandates process review, or changes the safety culture. Research should describe not only the list of components, but also how they were implemented, who delivered them, and which organizational changes occurred concurrently.

### 5.2. One Protocol for Every Patient?

Standardization reduces omissions, but a uniform pathway may be inappropriate for patients with severe frailty, shock, urgent surgery, complex aortic disease, or circulatory support. A reasonable objective is a common core with adaptive modules. Personalization should be based on predefined variables and should not become unjustified variation.

An adaptive pathway should also reflect patient-defined goals: identical procedural risks may lead to different acceptable choices when individuals place different weight on symptom relief, independence, cognitive recovery, or the burden of rehabilitation. Shared decisions require transparent communication of uncertainty and alternatives, not simply obtaining consent for a predefined plan.

Most studies come from experienced centers and enroll patients undergoing elective surgery. Generalization to hospitals with different resources and to high-risk patients requires pragmatic studies. Equity must also be assessed: a program dependent on devices, applications, or home support may exclude patients with lower digital literacy or less social support.

### 5.3. Are the Appropriate Outcomes Being Measured?

Length of stay is sensitive to care processes but strongly influenced by organization and social support. Mortality and major complications are important, although they require large samples. Future studies should incorporate a core outcome set including functional recovery, quality of life, PROMs, days alive and at home, readmissions, caregiver burden, and costs. Follow-up should extend beyond discharge.

Safety should be reported with the same level of detail as efficacy. A strategy may shorten length of stay while increasing hypotension, pain, reintubation, or unplanned health-care encounters. Reporting only favorable outcomes makes it difficult to determine which components should be generalized.

### 5.4. Balancing Accelerated Recovery, Safety, and Resource Use

Accelerated recovery is a means rather than an endpoint. Protocols should balance shorter ventilation and admission against reintubation, delayed recognition of low cardiac output, pain limiting mobilization, premature device removal, missed complications, and unplanned readmission. Conversely, unnecessary delays in extubation, mobilization, or discharge may expose patients to immobility, hospital-acquired complications, and avoidable resource use. Evaluation should therefore jointly report process measures, safety events, functional recovery, treatment delays, unplanned health-care encounters, and costs. Clinical exceptions should be documented rather than misclassified as protocol failure; the appropriate comparator is a well-implemented contemporary ERAS pathway, not an obsolete fast-track strategy.

### 5.5. Priorities

Priorities include comparing complete programs described in reproducible detail; measuring adherence by component; including frail and high-risk populations; establishing patient-centered outcomes; assessing cost-effectiveness and equity; and testing emerging interventions in impact studies rather than prediction studies alone. Perfusion and NIRS research requires standardized algorithms and clinically relevant outcomes. Prehabilitation studies should define selection, dose, and minimum duration. Regional analgesia requires head-to-head comparisons and safety surveillance. Delirium research should harmonize definitions and assessment periods.

## 6. Future Directions: Precision ERAS

In this review, precision ERAS is proposed as a structured and testable extension of contemporary ERAS care, in which predefined patient characteristics, informed preferences, and observed perioperative responses are linked to explicit adaptation rules. Its potential contribution is not individualization itself, which is already part of established perioperative medicine, but the reproducibility, auditability, and prospective evaluation of those adaptations. Clinical superiority over well-implemented conventional ERAS has not been demonstrated and must be assessed using patient-centered outcomes, safety measures, equity, and resource utilization.

Personalization does not mean abandoning standardization. A precision model would retain a core set of reliable practices while adapting the intensity of prehabilitation, monitoring, analgesia, PBM, rehabilitation, and follow-up according to risk and response. Assessment should combine clinical variables, function, frailty, preferences, and social context.

Artificial intelligence models may improve risk discrimination, but a higher area under the curve does not demonstrate clinical utility. Before implementation, calibration, external validation, bias, explainability, integration into clinical workflow, and the consequences of acting on predictions must be evaluated. The same applies to biomarkers and wearable devices: their value lies in changing decisions safely and improving outcomes, not in increasing the volume of data.

Patient preferences and shared decisions are essential inputs to such adaptation. Precision should not be understood solely as algorithmic risk prediction; the choice of intervention and the definition of successful recovery must also be concordant with informed patient goals.

**Figure 3:**
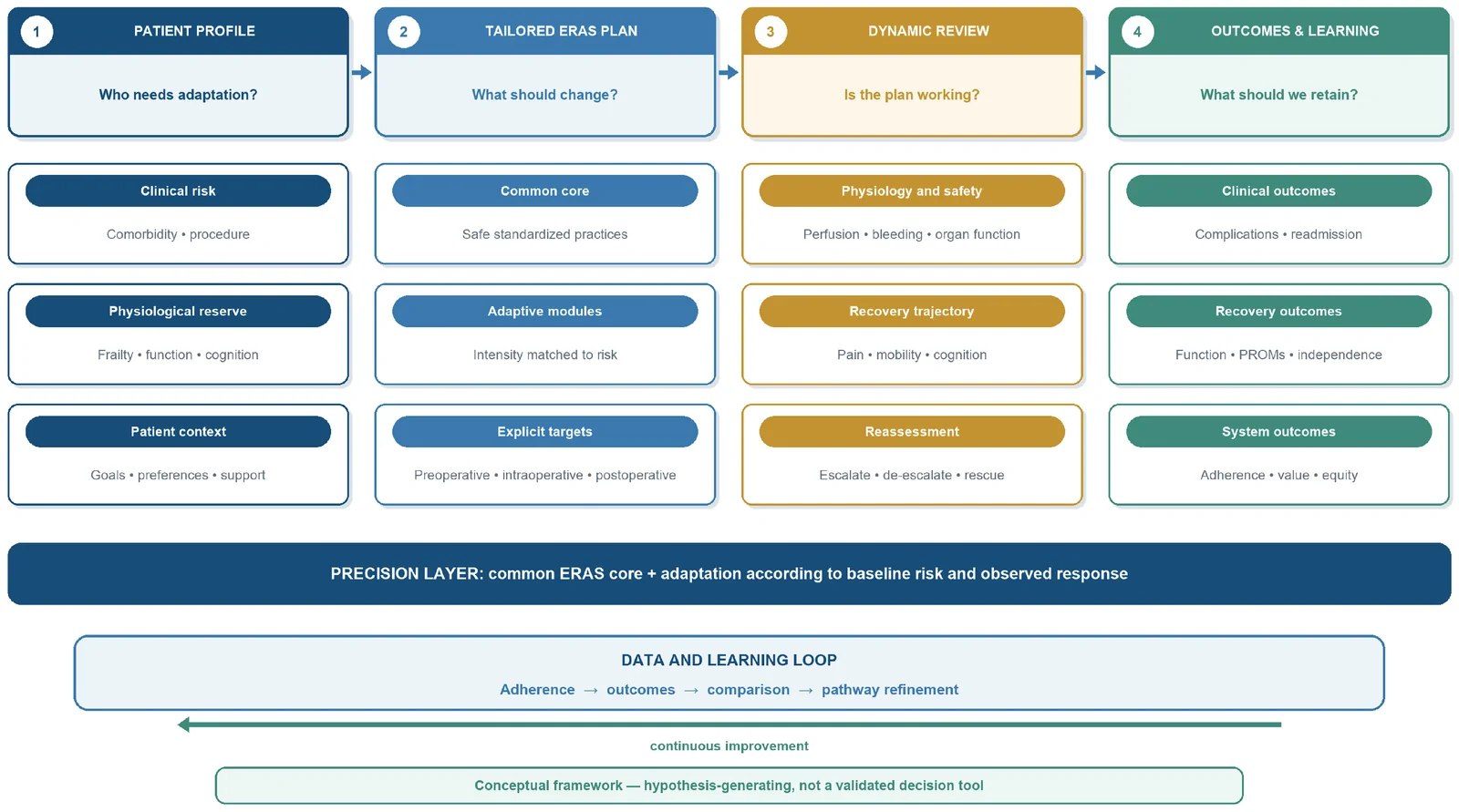
Precision Cardiac ERAS Framework. Conceptual proposal integrating risk stratification, a tailored perioperative plan, dynamic monitoring, patient-centered outcomes, and continuous learning. The framework has not been validated and should be regarded as hypothesis-generating.

Figure 3 presents the Precision Cardiac ERAS Framework as a conceptual proposal rather than a validated tool. It organizes a cycle of risk stratification, a tailored plan, monitoring, outcomes, and learning. Future evaluation would require predefined decision rules, comparators, outcomes, and data-governance mechanisms.

Three steps should be distinguished: (1) risk identification, in which a model or assessment predicts an outcome; (2) risk modification, in which an actionable intervention is selected on the basis of that assessment; and (3) demonstrated clinical benefit, which requires evidence that the assessment-guided decision improves patient-important outcomes compared with usual care. Discrimination and calibration alone establish neither treatment responsiveness nor clinical utility. Impact studies should test predefined decision thresholds, management changes, harms, equity, and net benefit.

The illustrative hypothetical decision pathway (not validated): Consider a 79-year-old patient undergoing elective valve surgery with slow gait speed, chronic kidney disease, and a stated priority to return home independently. Both a high-quality conventional ERAS pathway and the proposed precision approach would provide guideline-based assessment, shared decision-making, blood management, multimodal analgesia, safety-based extubation, delirium prevention, and early mobilization. The proposed framework would make three adaptation rules explicit: (1) if frailty screening indicates reduced functional reserve, the multidisciplinary team documents whether time and clinical stability permit targeted prehabilitation and plans an early physiotherapy assessment; (2) if renal vulnerability is identified, perfusion, fluid, nephrotoxic medication, and postoperative renal surveillance plans are documented and reassessed when clinical status changes, without imposing an unvalidated universal threshold; and (3) if the patient prioritizes independent living, discharge readiness and post-discharge rehabilitation intensity are assessed against mobility and activities of daily living goals, with escalation if recovery falls short. Each trigger, chosen action, clinical exception, and subsequent outcome would be recorded. Evaluation should compare this rule-based approach with well-implemented conventional ERAS using functional independence, acute kidney injury, days alive and at home, readmissions, patient-reported recovery, adverse events, and resource use. This is a hypothetical specification of testable decisions, not a validated algorithm or evidence of superiority.

## 7. Conclusions

ERAS has shifted attention from the isolated procedure to the complete recovery pathway. The available evidence primarily supports a modest reduction in length of stay and suggests benefits in complications and function, but certainty remains limited by heterogeneity, predominance of single-center studies, and evaluation of non-equivalent bundles. It has not been demonstrated that every protocol labeled ERAS produces the same outcomes.

Components of greatest practical relevance include assessment of physiological and nutritional reserve, optimization of anemia and Patient Blood Management, multimodal analgesia, physiologically guided perfusion, delirium prevention, structured mobilization, and an active transition to home. For each, the clinical objective should be distinguished from a specific technique, and the false precision of universal thresholds should be avoided. NIRS, wearables, and other technologies may complement assessment, but their usefulness depends on the context, response algorithm, accessibility, and quality of evidence.

The immediate priority is high-quality implementation: an accountable team, a common core, explicit adaptation, measurable adherence, and safety and recovery outcomes. Evolution toward precision perioperative medicine is plausible, but it must demonstrate that personalized decisions improve relevant outcomes, are equitable, and add value. Until then, progress will depend less on accumulating interventions than on selecting, coordinating, and evaluating existing ones more effectively.

## Figures and Tables

**Table 1:** Concise evidence map for major domains within enhanced recovery pathways in cardiac surgery.

Domain	Key Contemporary Evidence	Evidence Synthesis	Clinical Interpretation	Study Design	Certainty and Applicability (Narrative Appraisal)
Complete ERAS pathways	Randomized trials and meta-analyses [6,7,8,9]	Modest reductions in ICU and hospital stay and possibly fewer overall complications; no demonstrated mortality benefit.	Effects belong to the bundle and its delivery. Heterogeneity and implementation differences prevent attribution to individual measures.	RCTs and mixed-design meta-analyses	Low to very low for program-level outcomes; bundles and settings vary
Education, baseline reserve, and prehabilitation	Education meta-analysis, frailty cohort/meta-analysis, and prehabilitation meta-analysis [11,12,13,14,15]	Education improves knowledge and anxiety-related outcomes; frailty is prognostic; exercise-based prehabilitation may improve walking distance and shorten stay.	Assessment and education add value only when they change preparation and participation. Prehabilitation evidence remains small and heterogeneous.	RCT meta-analysis; observational cohorts; small trials	Education: variable; frailty: prognostic evidence; prehabilitation: limited, heterogeneous
Nutrition and metabolic preparation	Retrospective nutrition cohort and randomized carbohydrate-loading trials [16,17,18,19]	MUST independently predicted postoperative ADL decline; carbohydrate loading has inconsistent metabolic and clinical effects.	Screening is prognostic, not proof of treatment benefit. Link risk to a feasible intervention and individualize carbohydrate use.	Retrospective cohort; small RCTs	Low/uncertain for clinical benefit; carbohydrate trials largely elective
Anemia and Patient Blood Management	Cohort, treatment trial, PBM guideline, TITRe2, and meta-analysis [20,21,22,23,24,25]	Anemia is associated with AKI and longer stay; bundled hematinic treatment can reduce transfusion; no single transfusion threshold suits every patient.	Treat the cause and distinguish combination-treatment evidence from the independent effect of iron or erythropoietin.	Observational cohort; RCTs; guideline; meta-analysis	Component-specific; strong trial evidence for some transfusion strategies, no universal threshold
Analgesia and regional techniques	Randomized multimodal trial and parasternal-block meta-analysis [26,27]	Multimodal analgesia improves pain and nausea; parasternal blocks modestly reduce early opioid consumption.	Evidence does not establish a universal drug combination or superior block. Prioritize dynamic analgesia, safety, and recovery goals.	RCT; meta-analysis of RCTs	Short-term analgesic/opioid effects supported; technique comparison and safety uncertain
CPB perfusion and organ protection	DO_2_-guided trials, CPB guideline, and NIRS meta-analysis [28,29,30,31]	DO_2_-guided perfusion reduces postoperative AKI in selected protocols; NIRS-guided algorithms may reduce delirium.	Thresholds and response algorithms are context-dependent; mortality and long-term benefits remain unproven.	RCTs; clinical guideline; meta-analysis	AKI reduction in selected DO_2_ protocols; NIRS clinical benefit uncertain beyond delirium signal
Ventilation, glycemic control, and fluids	PROVECS, GLUCO-CABG, and LICRA trials [32,33,34]	Open-lung ventilation, intensive glucose targets, and chloride restriction did not improve their principal clinical outcomes.	Avoid converting plausible physiology into universal bundles or isolated numeric targets.	Multicenter randomized trials	Specific tested intensified strategies neutral; not evidence against basic protective care
Hemostasis and blood conservation	ATACAS and multicenter point-of-care algorithm trial [35,36]	Tranexamic acid reduces bleeding/transfusion but increases seizures; algorithmic point-of-care testing reduces platelet transfusion and major bleeding.	Dose, patient risk, defect-directed treatment, and safety outcomes must accompany blood-conservation metrics.	Large RCT; stepped-wedge cluster RCT	Trial-supported effects on bleeding/transfusion; seizures and safety require attention
Delirium, oral recovery, devices, and mobilization	Delirium trials/meta-analyses, hydration and chest-tube trials, and mobilization meta-analysis [37,38,39,40,41,42]	Drug evidence for delirium is mixed; early hydration improves comfort; very early drain removal is not automatically beneficial; mobilization improves function.	Use readiness, swallowing and safety criteria, multicomponent prevention, and functional outcomes rather than isolated milestones.	RCTs; systematic reviews/meta-analyses	Variable by intervention; functional effects and long-term safety less certain
Delivery system and transition home	Nurse-led follow-up, hybrid rehabilitation, pilot mHealth, and targeted rhythm-monitoring trials [43,44,45,46]	Structured follow-up can improve early symptoms, function, and satisfaction; targeted monitoring markedly increases AF detection.	Evidence for reduced readmission or mortality remains insufficient. Technology requires accessibility and an explicit escalation pathway.	Small RCTs; pilot RCT; monitoring RCT	Limited for readmission/mortality; monitoring detects AF, clinical benefit not established

Abbreviations: AKI, acute kidney injury; CPB, cardiopulmonary bypass; DO_2_, oxygen delivery; ERAS, Enhanced Recovery After Surgery; ICU, intensive care unit; NIRS, near-infrared spectroscopy; PBM, Patient Blood Management. Evidence is described by study design rather than converted into a formal GRADE rating because this is a narrative review.

## Data Availability

No new data were created or analyzed in this study. Data sharing is not applicable to this article.
